# Effects of response format on achievement and aptitude assessment results: multi-level random effects meta-analyses

**DOI:** 10.1098/rsos.220456

**Published:** 2023-05-03

**Authors:** Sonja Breuer, Thomas Scherndl, Tuulia M. Ortner

**Affiliations:** Division of Psychological Assessment, Department of Psychology, Paris Lodron University, Salzburg, Austria

**Keywords:** response format, achievement and aptitude assessment, open-ended, closed-ended, meta-analysis

## Abstract

Psychological achievement and aptitude tests are fundamental elements of the everyday school, academic and professional lives of students, instructors, job applicants, researchers and policymakers. In line with growing demands for fair psychological assessment tools, we aimed to identify psychometric features of tests, test situations and test-taker characteristics that may contribute to the emergence of test bias. Multi-level random effects meta-analyses were conducted to estimate mean effect sizes for differences and relations between scores from achievement or aptitude measures with open-ended (OE) versus closed-ended (CE) response formats. Results from 102 primary studies with 392 effect sizes revealed positive relations between CE and OE assessments (mean *r* = 0.67, 95% CI [0.57; 0.76]), with negative pooled effect sizes for the difference between the two response formats (mean *d*_av_ = −0.65; 95% CI [−0.78; −0.53]). Significantly higher scores were obtained on CE exams. Stem-equivalency of items, low-stakes test situations, written short answer OE question types, studies conducted outside the United States and before the year 2000, and test-takers' achievement motivation and sex were at least partially associated with smaller differences and/or larger relations between scores from OE and CE formats. Limitations and the results’ implications for practitioners in achievement and aptitude testing are discussed.

## 1. Effects of response format on achievement and aptitude assessment results: multi-level random effects meta-analyses

Standardized achievement and aptitude tests are basic elements of day-to-day life in educational, academic and professional settings around the globe. Whereas ‘“achievement” typically refers to knowledge and skills that are formally taught in academic settings’, ‘“aptitude” refers to an individual's characteristics that indicate the potential to develop a culturally valued ability’ [1, p. 2]. Beginning at the primary education level, achievement tests are used to determine whether a student is ready to pass a grade level and be promoted to the next. When leaving educational institutions, exit examinations need to be passed in many countries to receive a diploma or certificate (e.g. Finland, Germany, the United Kingdom, the United States; [2,3]). Subsequently, aptitude tests (often called entrance examinations) are commonly required for admission to secondary schools, post-secondary education such as colleges and universities, apprenticeships and professional careers. The use of aptitude and achievement tests that are fair, reliable and valid measures of relevant abilities and skills is of utmost importance, especially in selection situations, as rejected candidates may face negative consequences in terms of their academic futures and the attainment of professional and personal goals [4].

Given that achievement and aptitude tests aim to assess test-takers' maximum performance, systematically impaired performance due to construct-irrelevant sources of score variance may diminish the validity and fairness of such tests (e.g. [5–7]). Individuals’ test performance can be validly compared when scores have the same psychological meaning across test-takers [8], whereas test bias can be understood as systematic error that differentially diminishes test validity depending on individuals' group membership [9]. Dorans & Cook [10] defined test fairness as one of the essential psychometric standards for designing, developing and administering psychological assessments, and the Standards for Educational and Psychological Testing by the American Educational Research Association (AERA), the American Psychological Association (APA), and the National Council on Measurement in Education (NCME) have stated that ‘fairness is a fundamental validity issue and requires attention throughout all stages of test development and use’ [11, p. 49]. In her individual-differences (HID) model [12], Helms argued that systematic variance in test performance stemming from individuals' psychological characteristics that are irrelevant to the measured construct needs to be identified and removed in order to improve test fairness. Thus, identifying psychometric features of tests, environmental properties of test situations and test-taker characteristics that may contribute to the emergence of test bias is a highly relevant mission for psychological research.

Various response formats have been used in achievement and aptitude testing in the last couple of decades of testing practice. Probably the most popular response format in performance assessment is the closed-ended (CE) format, more commonly known as multiple-choice testing. CE test items consist of a stem (i.e. the question) and usually four or five simultaneously presented response options, of which one or more are correct. Depending on the number of alternatives and the number of potentially correct options, a certain guessing probability (i.e. the likelihood of choosing the correct response(s) by chance) exists in CE testing. So-called *true–false* items or questions with only two response options, one of which is correct, provide the highest guessing probability of 50%. The guessing probabilities for other common CE formats, such as single-choice items with four or five response alternatives or single-choice questions with 6 to 10 options, one of which is correct, are 20–25% and 10–16.7%, respectively. By using multiple-choice items with four or five response alternatives, one or more of which are correct, the guessing odds can be reduced to 6.3% or even 3.1%, respectively. Various types of open-ended (OE) response formats, often also called constructed-response or free-response formats, are also commonly used in achievement and aptitude testing. These include *written short answer, cloze, essay, oral OE* and *practical task* items, which require the test-taker to write a brief response, fill in a blank, write a short composition, react verbally to questions, or demonstrate their skills and knowledge in hands-on applications, respectively.

There is a long history of discussion about the pros and cons of CE over OE response formats in performance assessment (e.g. [13–18]). Test administrators value the objectivity, economy and efficiency of scoring in CE testing (e.g. [19]), and test-takers often perceive CE items as easier (e.g. [20]). On the other hand, critics argue that higher-level cognitive processes are required to answer OE test items, whereas on CE tests, retrieving learned facts and sources other than learning success or high cognitive performance can contribute to a high score, such as test-wiseness strategies [21–24]. Even though relatively high uncorrected correlation coefficients have been found for test performance in assessments with OE and CE response formats (e.g. 0.67; [25]), prior research has questioned the construct equivalence of OE and CE test items (e.g. [26–28]) and some variance based on response formats still needs to be empirically explained [13,25,29,30]. The possibility that an exam in one response format can yield significantly different scores on the same exam in another response format raises doubts concerning the construct equivalence of the OE and CE assessment formats.

Consequently, with the present work, we aimed to meta-analytically integrate existing research findings on the construct equivalence of test scores based on OE and CE response formats. Furthermore, we aimed to identify potential moderating effects of (i) test features rooted in the examination situation and (ii) test-takers' individual characteristics. Previous literature reviews and meta-analyses addressing the relations between scores from OE and CE response formats have been conducted by Traub [31], Ryan & DeMark [32], Rodriguez [25] and In'nami & Koizumi [33]. Traub [31] and Rodriguez [25] included nine and 67 primary studies in their meta-analyses, respectively, and focused mainly on correlations between scores from OE and CE response formats. Traub [31] concluded that the number of studies was too small to validly assess construct equivalence. He cautiously inferred that the two formats measured slightly different constructs in the writing and word knowledge domains. For reading comprehension and quantitative domains, on the other hand, he suggested that the two formats assessed similar constructs. Rodriguez [25] identified an uncorrected pooled correlation coefficient of 0.67 and concluded that construct equivalence appeared to be at least partly a function of item design (e.g. stem-equivalency; [25]). Ryan & DeMark [32] conducted two meta-analytic studies examining score differences between men and women in relation to response format. The first meta-analysis included 14 primary studies with tests of language, mathematics, science and social studies, whereas the second meta-analysis specifically examined language and mathematics assessments and included 23 additional primary studies. Their analyses revealed no or small differences between men's and women's scores in relation to the response format in science, social studies and mathematics (i.e. Cohen's *d* < 0.20). With respect to language assessments, effect sizes of −0.25 to −0.30 indicated that women slightly outperformed men when OE formats were employed. The results of In'nami & Koizumi's [33] most recent meta-analysis on format effects on reading and listening test performance included 37 primary studies and indicated that CE formats were easier for test-takers than OE formats (Hedges' *g* = 0.65), especially when between-subjects designs or stem-equivalent items were used. However, previous meta-analyses have had some limitations concerning the effects of response format on achievement and aptitude assessment results. For example, Rodriguez [25] focused exclusively on correlations, entirely excluding studies that examined difference hypotheses. Ryan & DeMark [32] as well as In'nami & Koizumi [33] restricted their work to mathematics and language ability only. Thus, as the most recent meta-analysis was conducted over a decade ago and all previous findings were rather inconclusive, we did not try to replicate earlier meta-analyses, but aimed to quantitatively synthesize all available information about response format effects in a much more detailed way. Furthermore, we aimed to include a larger number of potential moderators than prior research.

In the literature, several potential moderating effects of test design characteristics have been reported to be relevant for scores on tests with different response formats. Some authors have come to the conclusion that construct equivalence between assessments in different response formats is given when stem-equivalent items are used, that is, when the only difference between the OE and CE version of an item is that the latter provides response options (e.g. [25,33]). Potentially performance-decreasing or performance-enhancing factors, such as test anxiety or achievement motivation, are considered to have a higher impact in high-stakes test situations with potentially dramatic consequences for individuals than in low-stakes situations, when the test results are not personally important for individuals (e.g. [34,35]). These performance-influencing factors, in turn, are widely known to interact with response formats (e.g. [36,37]; further discussion follows in the next section). In previous research (as reviewed by [13,25,29]), scores obtained from various OE response formats (e.g. *cloze*, *written short answer*, *essay*) and CE response formats (e.g. *true–false*, *single-choice*, *multiple-choice* items) in within- and between-designs and in small-scale as well as large-scale assessment conditions have been empirically compared within several domains (e.g. mathematics, reading comprehension, vocabulary). Scores from OE formats requiring a short response similar to typical response options in CE exams (i.e. *written short answer*) have generally yielded stronger relations with scores from CE modes than OE formats requiring longer responses (e.g. essay types; [25,38]). Among CE response formats, a reduced guessing probability due to a larger number of response alternatives and correct options has been found to lead to smaller differences in scores between CE and OE assessments (e.g. [39]).

In addition to these moderating effects of test design, a variety of individual test-taker characteristics have been proposed to interact with response format, including test anxiety, risk propensity, achievement motivation, sex and age. Test anxiety, defined as an extreme fear of being negatively evaluated on upcoming tests [40], has been acknowledged as a source of bias and underperformance in standardized testing (as reviewed by Zeidner [40] and McDonald [41]). With respect to response format, test-takers with higher levels of anxiety have been hypothesized to perform relatively better on CE assessments than on OE assessments, as CE tests are considered to be less frightening [40,42]. Furthermore, the distracting impact of test-related worries may make it more difficult to construct a correct response than to merely recognize it [36,43]. A second personality aspect commonly set in relation to response format is risk propensity, defined as the willingness to exhibit behaviour that involves an unknown probability of danger and negative consequences but also the possibility of gaining advantages or benefits [44]. Many studies have found that test-takers with higher levels of risk propensity are significantly more likely to guess in CE response formats, whereas people with lower risk propensity prefer to skip questions when they are unsure about the correct solution [26,45,46]. Additionally, achievement motivation (i.e. the need to master difficult tasks and improve one's performance relative to some standard of excellence, as defined by Edgerton & Roberts [47]) has been mentioned as relevant. High levels of achievement motivation have been found to be positively related to the effective use of test-taking strategies [48], which, in turn, are known to be especially performance-enhancing in assessments with a CE response format (e.g. [13,49]).

All three of these aspects of personality—test anxiety, risk propensity and achievement motivation—are supposed to interact not only with response format but also with sex (e.g. [32,50–55]) and age (e.g. [41,50,56,57]). Data analyses have revealed that men often outperform women on CE assessments, whereas both sexes perform approximately equally on OE exams, and sometimes women perform even better (e.g. [32,58,59]). Studies have furthermore revealed that men tend to guess more often and therefore score higher on CE tests, whereas women tend to omit more items [60–62]. In terms of the life course, these individual characteristics that potentially interact with test format usually begin to differentiate during adolescence (e.g. [41,57,62,63]). This trend has been interpreted as suggesting that differences in potential response format effects caused by test anxiety, risk propensity or achievement motivation might grow in magnitude with increasing age.

In summary, the literature indicates a consensus among research that scores on aptitude and achievement tests with OE and CE response formats are typically highly related [25]. Nevertheless, the magnitudes of these interrelations have been found to fluctuate substantially (e.g. [64–66]). Consequently, there is further need to evaluate the effects of response format on scores from different response modes. The first purpose of the present work was, therefore, to meta-analytically summarize the existing research findings on the differences and relations between scores from OE and CE response formats for achievement and aptitude measures. Second, we studied moderating effects of the examination situation and individual characteristics of the test-takers. On the basis of prior research findings, we hypothesized that a medium to large positive relation between CE and OE scores would reveal, because the original studies aimed to measure the same constructs with these two response formats. Nevertheless, we expected higher scores to be achieved on CE tests on average, due to the score-enhancing benefits of guessing probability and the need to simply recognize one of the response options, as opposed to having to recall a solution on OE examinations [33]. Furthermore, we were interested in whether larger relations between scores from OE and CE assessments would coincide with smaller standardized mean differences between scores from the two formats.

Regarding the moderating effects of examination situations, we hypothesized a smaller difference and stronger relation between scores from CE and OE formats (i) when stem-equivalent items were used (e.g. [25,33]), (ii) in low-stakes test situations, because impairing factors (e.g. test anxiety) should have stronger effects on performance when the test can have dramatic consequences for test-takers (e.g. [34,35]), and (iii) when *written short answer* items were used rather than other OE response types, because the former are usually more similar to typical CE item formats (e.g. [25,38]). Furthermore, we presumed smaller differences and stronger relations between scores from OE and CE items as the guessing probability dropped (in descending order: *true–false*, *single-choice 1 out of 4 or 5 options*, *single-choice 1 out of 6 to 10 options* and *multiple-choice x out of 4 or 5 options*), as proposed by Kubinger & Gottschall [39]. Additionally, we aimed to examine the potential moderating effects of the study design, the country in which the original study was conducted, the year of data collection and the study scope (i.e. small-scale versus large-scale) in exploratory analyses.

Concerning the moderating effects of test-takers' individual characteristics, we hypothesized that the difference between scores from OE and CE response formats would increase as test-taker age increased (e.g. [41,57,63]) and assumed that larger sex differences in favour of men would arise in CE formats, whereas smaller sex differences or even a bias favouring women would arise in OE formats, as men have been shown to be more prone to guessing and successfully using test-taking strategies in examination situations (e.g. [32,58,59]). We presumed that test anxiety would be more strongly linked to performance on OE items than on CE items, because CE tests have been shown to provoke less anxiety, and test anxiety has been shown to particularly interfere with working memory in tasks requiring recall rather than recognition (e.g. [40,42]). Both risk propensity and motivation have been hypothesized to be more strongly related to performance on CE items than OE items, because test-takers who are more prone to taking risks tend to guess more often (e.g. [26,45,46]), whereas motivation has been linked to better use of test-taking strategies (e.g. [48]). Finally, alongside the moderating effects of individual characteristics, we aimed to find out whether scores obtained from OE versus CE examinations would be differentially related to school performance.

## 2. Method

The meta-analyses were conducted in accordance with the APA's *meta-analysis reporting standards* (MARS; [67]), the *PRISMA Statement* (preferred reporting items for systematic reviews and meta-analyses; [68]) and practical recommendations for improving the reproducibility of meta-analyses [69]. Datafiles, R codes and codebook for this meta-analysis are made available in the Open Science Framework (https://osf.io/vry9f/?view_only=fca6caab4b3341cb9b5b6dccfa576859).

### 2.1. Literature search, inclusion criteria and exclusion criteria

We included two electronic databases (Web of Science with all citation indices and PsycINFO) in our search for relevant literature, applying the search string ((*response format or test format or item format*) *or* ((*multiple choice or multiple-choice or forced choice or forced-choice or multiple select or multiple-select or single choice or single-choice or closed format or true–false*) *and* (*free response or constructed response or open format or essay or short answer or open ended*))) *and* (test* or perform* or exam* or abilit* or achiev* or skill* or scor*) *and* (gender or sex or age or personal* or risk or anxiety or extraversion or openness or conscientiousness or agreeableness or neuroticism) from inception to 1 September 2022. Additionally, we hand-searched the reference lists of relevant publications for studies not provided by the databases in response to the search string. Furthermore, we contacted relevant authors for unpublished data and included all data received by 2 November 2022.

The studies were required to meet our inclusion criteria (for details, table 1) with reference to participants (healthy participants of all ages), intervention (cognitive performance assessment in different response formats), comparator (response format; i.e. OE versus CE), outcome (cognitive performance), study design (quantitative studies with an English-language abstract and extractable effect size) and setting (low-stakes and high-stakes test situations, large-scale and small-scale assessments). We included randomized and non-randomized studies with stem-equivalent and non-stem-equivalent items, conducted with either a between-design, in which participants took tests measuring the same construct in either an OE or a CE version, or a within-design, in which the same participants worked on items with different response formats. Accordingly, we excluded studies that included participants with cognitive impairments (e.g. due to agenesis of the corpus callosum; [70]) or mental health disorders (e.g. schizophrenia; [71]), studies that used outcomes other than cognitive performance (e.g. learning styles; [72]) and qualitative studies (e.g. [73]). Furthermore, we excluded scientific work comparing response formats other than OE versus CE (e.g. structured response format versus semi-structured interview; [74]), studies that did not present clearly separable effect sizes for the OE and CE parts of a test (e.g. scores comprising both OE and CE items; [75]), and studies in which the OE and CE parts did not measure exactly the same construct (e.g. [76]).

**Table 1.** PICOSS table showing the inclusion criteria for the meta-analyses.

participants	healthy participants of all ages
intervention	cognitive performance assessment in different response formats
comparators	response format (OE versus CE) and individual characteristics (e.g. sex, age, test anxiety, risk propensity)
outcome	scores on cognitive achievement and aptitude tests (e.g. psychometric cognitive ability tests, school and university exams, entrance tests, international student assessments)
study design	all quantitative studies with English-language abstract and extractable effect size, within- or between-design, stem-equivalent and non-stem-equivalent items, randomized and non-randomized studies
setting	high- and low-stakes test situations, large-scale and small-scale assessments

**Figure 1.**
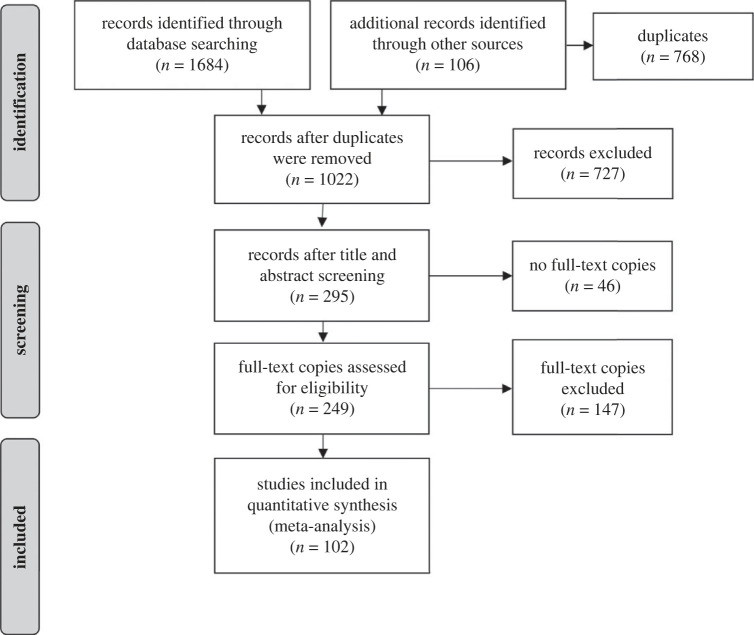
Flow chart for study identification and selection.

A total of 1790 records were identified through the database search and other sources by one rater (figure 1). After removing duplicates, 1022 titles and abstracts were screened by two raters (inter-rater reliability for 100 records: intraclass correlation coefficient (ICC) = 0.94). Full-text copies of 249 records were obtained and screened for eligibility by two raters (inter-rater reliability for 100 records: ICC = 0.91). Disagreements were resolved following discussion. Data from 102 records (marked with an asterisk in the Reference list) were extracted by two raters. Inter-rater reliability was perfect for the extracted sample sizes (ICC = 1.00, *k* = 163) and very high for the extracted effect sizes (ICC = 0.96, *k* = 163) in Meta-Analyses A, very high for the extracted sample sizes (ICC = 0.97, *k* = 38) and perfect for the extracted effect sizes (ICC = 1.00, *k* = 38) in Meta-Analyses B, and perfect for the extracted sample sizes (ICC = 1.00, *k* = 153) and extracted effect sizes (ICC = 1.00, *k* = 153) in Meta-Analyses C. All disagreements between raters concerning sample sizes and effect sizes were resolved via discussion.

### 2.2. Coding

Aside from the papers' names, authors, years, comparators and outcome details as stated in the PICOSS table (table 1), we extracted several other study and outcome characteristics, such as the country from which the sample was drawn, sample size, the mean age of the test-takers and the percentage of men and women in the sample, when reported. Additionally, we coded potential moderators, such as the stem-equivalency of the items, study design, the level of consequences for the test-taker, scope and school performance. Furthermore, for our moderation analyses, we extracted separate score means and standard deviations per response format for men and women, and correlations between individual test-taker characteristics (e.g. test anxiety, risk propensity, motivation) and test scores per response format. To structure the data, we clustered information about the respective papers’ response formats and test situations into global categories via discussions among the raters. A close examination of the descriptions revealed that all OE format types could be assigned to the categories *written short answer*, *cloze*, *essay*, *mixed OE*, *oral OE* and *practical task*. We successfully allocated most of the CE format types to the categories *single-choice*, *multiple-choice* and *true–false* with different numbers of response options (i.e. *x out of 4 or 5 options*, *1 out of 4 or 5 options*, *1 out of 6 to 10 options*). When no further information about the type of CE format was reported, the term *undefined CE* was used. The original articles employed more than 100 different aptitude and achievement tests (e.g. psychometric reasoning tests, school and university examinations, international student assessments) assessing various abilities (e.g. natural science knowledge, reading comprehension, maths ability). Therefore, we used the nomenclature from the revised Cattell–Horn–Carroll (CHC) model of cognitive abilities [77] to cluster the reported performance scores into the global categories G*rw* (reading and writing ability), G*kn* (domain-specific knowledge), G*q* (quantitative knowledge), G*f* (fluid reasoning), G*l* (learning efficiency) and G*v* (visual processing). Scores with negative polarity were recoded.

### 2.3. Data analysis

Most results included in the original studies were available as means or could be calculated into means (e.g. raw scores, percentages, proportion scores, item difficulties, *z*-scores), supplemented with standard deviations or standard errors, with the sample size and/or the number of items per response format also reported. Some results were presented as coefficients from statistical difference tests (e.g. *t* tests, ANOVAs), and others as correlation coefficients between scores from different response formats. Only a few of the original records reported differences or relations between scores from test formats with respect to individual characteristics (e.g. sex, test anxiety, risk propensity). As a result of these divergences in the reported results, we computed standardized mean differences (i.e. Cohen's *d*_av_ according to Lakens [78], as not all primary studies with within-design reported correlation coefficients for OE and CE format scores) with the formula *d*_av_ = *M*_diff_/((SD_1_ + SD_2_)/SD_pooled_)^1^ or via the *practical meta-analysis effect size calculator* (with a correction factor for small sample sizes to calculate Hedges' *g*; [79]) for the results from difference tests. We computed the variance for Cohen's *d*_av_ with the formula *Vd*_av_ = (*n*_1_ + *n*_2_)/(*n*_1_ × *n*_2_) + (*d*_av_^2^/2 × (*n*_1_ + *n*_2_)) and *r* for correlations. We followed Cohen's [80] guidelines for identifying *small* (*r* = 0.10, *d* = 0.20), *medium* (*r* = 0.30, *d* = 0.50) and *large* (*r* = 0.50, *d* = 0.80) effects. Furthermore, depending on the reported results, we calculated either Cohen's *d*_av_ or *r* as comparable combined effect sizes for the moderation analyses that addressed the effects of the individual characteristics. Therefore, the primary studies' results were meta-analytically integrated in three ways: (i) to test hypotheses about differences, (ii) to examine hypotheses about relations, and (iii) to calculate moderating effects of individual test-taker characteristics.

To prepare our coded data for further analyses, we calculated standard deviations from standard errors and combined means and standard deviations in cases in which such values were only reported separately by group (e.g. by sex, ethnicity). Whenever raw scores from varying response formats were presented on different scales, we combined the scores into the same scale by computing percentages. For studies that used a within-design, we decided to calculate Cohen's *d*_av_ as well because, according to Westfall, ‘one of the primary motivations for using standardized effect sizes at all is so that we can try to meaningfully compare effects from different studies, including studies that might use different designs. But all of the effect size candidates other than classical Cohen's *d* are affected by the experimental design; that is, the “same” effect will have a larger or smaller effect size based on whether we used a between- or within-subjects design’ [81, p. 1]. When coefficients of within-ANOVAs were reported without descriptive statistics, we computed eta-squared or partial eta-squared and transformed these to Cohen's *d*. To avoid distorting our pooled effect size estimates, we removed outliers (i.e. *d* > 3 and <−3; *r* > 0.90) because, in these cases, the original studies' confidence intervals did not overlap with the confidence interval for the total pooled effect size [82]. For the standardized mean differences in the first group of meta-analyses, a positive effect size indicated that higher average scores were reached in OE response formats, and a negative effect size revealed that higher average scores were obtained in CE formats. For the standardized mean differences in the third group of meta-analyses, a positive effect size indicated that men reached higher scores and a negative effect size indicated that women obtained higher scores. We conducted multi-level random effects meta-analyses using the packages *metafor* (v. 3.8-1; [83])*, metaviz* (v. 0.3.1; [84]) and *dmetar* (v. 0.0.9000; [85]) in R (v. 4.0.5; [86]) in order to address effects that resulted from the fact that many of the studies reported more than one relevant effect size and therefore violated the requirement of independence of observations. We modelled the nestedness of the data by using the *robust.rma.mv()* function from the *metafor* package and specified the random part of the model as *∼1|citation/id*. This function uses a robust sandwich-type estimator in a multi-level meta-analysis. Additionally, we used the R package *clubSandwich* [87] to obtain robust estimators for standard errors and confidence intervals. We chose the *bias-reduced linearization adjustment* (*CR2*) proposed by Bell & McCaffrey [88] and further developed in Pustejovsky & Tipton [89]. The adjustment is used so that the variance–covariance estimator is exactly unbiased under a user-specified working model. These two robust estimations yielded almost identical results. Therefore, we report the results of the *robust.rma.mv()* function throughout the paper, whereas the *clubSandwich* estimators can be found in the markdown in the OSF repository. Cochran's *Q* and *I*^2^ were calculated to assess statistical heterogeneity. Cochran's *Q* checked whether the amount of variability in the studies' outcomes was statistically significant, whereas *I*^2^ quantified the extent of heterogeneity. We furthermore divided $I_{\mathrm{total}}^{2}$ into variability within studies $\left(I_{\mathrm{level}2}^{2}\right)$ and between studies $\left(I_{\mathrm{level}3}^{2}\right)$ to identify the level on which the heterogeneity originated. Following the guidelines of the *Cochrane handbook for systematic reviews of interventions* (v. 6.3; [90]), *I*^2^ values between 30% and 60% were interpreted as moderate heterogeneity, whereas *I*^2^ values above 75% suggested considerable heterogeneity.

In the first group of meta-analyses, we estimated overall pooled effect sizes for the difference in performance scores stemming from varying response formats and pooled effect sizes per area of cognitive ability when more than five effect sizes were extracted for each response format. Furthermore, we examined the moderating effects of eight variables related to the examination situation: (i) the items’ stem-equivalency (i.e. stem-equivalent versus non-stem-equivalent), (ii) the level of consequences for the test-taker (i.e. high-stakes versus low-stakes testing situation), (iii) type of OE format (i.e. *written short answer*, *cloze*, *essay*, *mixed OE*, *oral OE*, *practical task*), (iv) type of CE format including different numbers of response options (i.e. *true–false*, *single-choice 1 out of 4 or 5 options*, *single-choice 1 out of 6 to 10 options*, *multiple-choice x out of 4 or 5 options*), (v) time of data collection (i.e. before the year 2000 versus in or after the year 2000), (vi) country, (vii) design (i.e. within- versus between-studies), and (viii) study scope (i.e. small-scale versus large-scale assessment). As there were an insufficient number of effect sizes per group to statistically estimate the effects in every single category separately for each area of cognitive ability, we aggregated the type of OE format into two categories (i.e. *written short answer* versus *other OE types*), the type of CE format into two categories (i.e. *single-choice* versus *multiple-choice*), and the country into two categories (i.e. the United States versus other countries) for the purpose of the moderation analyses. In order to check for potential publication bias (i.e. the phenomenon that studies with significant results are more likely to be published than studies without significant outcomes), we computed Kendall's Tau (i.e. rank correlation between Cohen's *d*_av_ and sample size; [91]) and Egger's regression test [92]. We used the recommendations and formulae given by Fernández-Castilla *et al.* [93] to account for the nestedness of the data in the correlations between Cohen's *d*_av_ and its variance. Significant Kendall's Tau and Egger's coefficients indicate funnel asymmetry, which may be caused (among other reasons) by publication bias.

In our second group of meta-analyses, we estimated overall pooled effect sizes for the relations between scores from aptitude and achievement tests with OE and CE response formats, respectively, and pooled effect sizes per area of cognitive ability. We decided to compute meta-analyses for cognitive ability areas with more than five individual effect sizes because a smaller number of studies often led to extreme heterogeneity and very low power. Additionally, we calculated moderator analyses for the same eight variables related to the examination situation, as in the first group of meta-analyses. To investigate whether larger relations between scores from OE and CE assessments would coincide with smaller standardized mean differences between scores from the two formats, we calculated Pearson's product-moment correlation coefficients between effects available as both standardized mean differences and as correlation coefficients.

Finally, in our third group of meta-analyses, we examined moderating effects of the test-takers' individual characteristics when more than four effect sizes could be extracted per variable. We did not compute any meta-regression models with multiple moderators because the number of studies in the cells (especially when categorical moderators were used) was small, and thus, the power tended to get quite low. Additionally, the combination of multiple predictors relies on the assumption of uncorrelated (weakly correlated) predictors. Because of the sparse amount of data in the literature (many studies did not report correlations between the moderators), we felt that this assumption might or might not hold. On the basis of this doubt, we decided to stick to simple models (i.e. those that used only one moderator at a time). Most of the original studies did not report the test-takers' exact ages. However, they reported test-takers' school grade or phase of university studies, so we derived country-specific approximate age groups from this information. We decided to divide the test-takers into age groups that correspond to educational levels common in most countries, (i.e. 1 = preschool, under-6-year-olds; 2 = primary school, 6-to-10-year-olds; 3 = lower secondary school, 11-to-14-year-olds; 4 = upper secondary school, 15-to-17-year-olds; 5 = university students and adults, over-18-year-olds). More than four effect sizes were available for the categorical variables age group and sex (self-reported: male versus female) and the continuous variables test anxiety, risk propensity and achievement motivation. Furthermore, we calculated whether scores obtained on OE versus CE examinations were differentially correlated with school performance.

## 3. Results

### 3.1. Results of Meta-Analyses A: differences in scores from OE and CE formats and moderating effects of the test situation

#### 3.1.1. Descriptive statistics for Meta-Analyses A

The results of 81 primary studies with 303 effect sizes were included in the first group of meta-analyses (see electronic supplementary material, tables S1 and S4). A large majority of the studies had been conducted in the United States (49.4%), the United Kingdom (6.2%), Canada (6.2%) and Taiwan (4.9%), usually with school students (54.3%) and university students (39.5%) as the test-takers. Most of the authors used a within-subject design (77.8%) with non-stem-equivalent items (61.7%) in low-stakes test situations (71.6%) and small-scale assessments (70.4%). About half of the data were collected before the year 2000 (44.4%), and the number of test-takers ranged from 15 to 191 040 per record. The achievement and aptitude tests that were employed usually measured *domain-specific knowledge* (46.2%; e.g. natural science, economics and history knowledge), *reading and writing* (24.3%; e.g. reading comprehension, language proficiency and vocabulary knowledge) and *quantitative knowledge* (23.1%; i.e. maths and statistics ability).

#### 3.1.2. Multi-level random effects Meta-Analyses A

The first group of multi-level random effects meta-analyses yielded a statistically significant medium to large negative pooled effect size for the difference between scores from OE and CE response formats across all cognitive ability areas (*d*_av_ = −0.65, *p* < 0.001; table 2; details are presented in electronic supplementary material, table S7), indicating that, overall, higher scores were obtained for CE assessments. In detail, this was the case for the ability categories G*kn* (domain-specific knowledge; *d*_av_ = −0.68, *p* < 0.001), G*q* (quantitative knowledge; *d*_av_ = −0.65, *p* < 0.001), G*rw* (reading and writing; *d*_av_ = −0.66, *p* < 0.001) and G*l* (learning efficiency; *d*_av_ = −0.64, *p* = 0.036), indicating that significantly higher performance was obtained in the original studies when domain-specific knowledge, quantitative knowledge, reading and writing or learning efficiency were assessed via examinations with a CE response format rather than with OE items. With respect to heterogeneity, considerable variability was revealed for all cognitive ability categories. The heterogeneity within studies was low overall as well as for the ability categories G*kn*, G*q* and G*l*, but it was moderate for G*rw*. Between studies, considerable variability was found for all ability categories. Significant Kendall's Tau rank correlation (*τ* = 0.30, *p* < 0.001) and Egger's regression test (*z* = −6.06, *p* < 0.001) coefficients indicated the possible presence of publication bias overall. When we repeated these analyses for the separate cognitive abilities, significant Kendall's Tau rank correlation coefficients were found for the ability categories G*kn* (*τ* = 0.30, *p* < 0.001) and G*q* (*τ* = 0.24, *p* < 0.001) but not for G*rw* (*τ* = 0.04, *p* = 0.699) or G*l* (*τ* = 0.50, *p* = 0.173). Taking the nestedness of the data into account, Egger's regression test coefficients suggested funnel asymmetry in the ability categories G*kn* (*z* = −4.57, *p* < 0.001), G*l* (*z* = −2.20, *p* = 0.028) and G*q* (*z* = −2.42, *p* = 0.016) but not in the ability category G*rw* (*z* = 0.18, *p* = 0.860; see also the contoured funnel plots in electronic supplementary material, figure S1).

**Table 2.** Results of multi-level random effects Meta-Analyses A: pooled effect sizes for the differences between scores from OE and CE response formats. *Note*. *k* = number of effect sizes extracted, cognitive ability: G*kn* = domain-specific knowledge, G*q* = quantitative knowledge, G*rw* = reading and writing, G*l* = learning efficiency. s.e. = standard error, 95% CI = 95% confidence interval, Cochran's *Q* and $I_{\mathrm{total}}^{2}$ = measures of heterogeneity, $I_{\mathrm{level}2}^{2}$ = heterogeneity within studies, $I_{\mathrm{level}3}^{2}$ = heterogeneity between studies. All *I*² are percentages.

cognitive ability	*k*	Cohen's *d*_av_ (s.e.)	95% CI	*p*-value	Cochran's *Q*	$I_{\mathrm{total}}^{2}$	$I_{\mathrm{level}2}^{2}$	$I_{\mathrm{level}3}^{2}$
total***	303	−0.65 (0.066)	[−0.78, −0.53]	<0.001	*Q*(302) = 159 858.74, *p* < 0.001	99.83	25.38	74.45
Gkn***	132	−0.68 (0.108)	[−0.89, −0.46]	<0.001	*Q*(131) = 83 613.32, *p* < 0.001	99.88	21.06	78.82
Gq***	117	−0.65 (0.123)	[−0.89, −0.41]	<0.001	*Q*(116) = 40 424.53, *p* < 0.001	99.81	18.07	81.74
Grw***	43	−0.66 (0.101)	[−0.85, −0.46]	<0.001	*Q*(42) = 19 169.37, *p* < 0.001	99.59	31.14	68.45
Gl*	6	−0.64 (0.305)	[−1.24, −0.04]	0.036	*Q*(5) = 25.65, *p* < 0.001	90.98	21.93	69.05

*p < 0.05, **p < 0.01, ***p < 0.001.

#### 3.1.3. Moderation analyses A

Turning to moderating effects of the examination situation, the items’ stem-equivalency (non-stem-equivalent versus stem-equivalent; table 3) significantly moderated the difference between scores from OE and CE response formats. Overall, analyses revealed a significantly smaller difference between scores from OE and CE formats for stem-equivalent items than for non-stem-equivalent items (*d*_diff_ = −0.36, *p* = 0.004), as hypothesized. In detail, test-takers achieved even higher scores on CE tests compared with OE exams when non-stem-equivalent items were used overall and in the cognitive ability category G*kn* (*d*_diff_ = −0.49, *p* = 0.017), whereas the difference between OE and CE scores was smaller for stem-equivalent items.

**Table 3.** Results of moderation analyses A. *Note*. *k*_1_ = number of effect sizes extracted for moderator category 1, *d*_1_ = Cohen's *d*_av_ coefficient for moderator category 1, *k*_2_ = number of effect sizes extracted for moderator category 2, *d*_2_ = Cohen's *d*_av_ coefficient for moderator category 2, *d*_diff_ = difference between *d*_1_ and *d*_2_, G*kn* = domain-specific knowledge, G*q* = quantitative knowledge, G*rw* = reading and writing, SA = short answer, SC = single-choice, MC = multiple-choice.

moderator category								
1	2	cognitive ability	*k*_1_	*d*_1_	*k*_2_	*d*_2_	*d*_diff_	*p*-value
moderator: stem-equivalency								
yes	no	total**	242	−0.52	61	−0.88	−0.36	0.004
yes	no	Gkn*	106	−0.54	26	−1.03	−0.49	0.017
yes	no	G*q*	105	−0.62	12	−0.70	−0.08	0.749
yes	no	G*rw*	30	−0.54	13	−0.88	−0.34	0.106
moderator: level of consequences of the test situation								
low-stakes	high-stakes	total	236	−0.71	67	−0.51	0.19	0.108
low-stakes	high-stakes	G*kn*	79	−0.75	53	−0.59	0.16	0.315
low-stakes	high-stakes	G*q*	112	−0.69	5	−0.24	0.45	0.098
low-stakes	high-stakes	G*rw*	34	−0.69	9	−0.52	0.17	0.534
moderator: type of OE response format								
written SA	other OE	total	120	−0.65	183	−0.66	−0.01	0.908
written SA	other OE	G*kn*	57	−0.72	75	−0.60	0.12	0.542
written SA	other OE	G*q*	25	−0.67	92	−0.60	0.07	0.770
written SA	other OE	G*rw*	32	−0.60	11	−0.81	−0.20	0.383
moderator: type of CE response format								
SC	MC	total	236	−0.67	67	−0.62	0.05	0.703
SC	MC	G*kn*	91	−0.69	41	−0.65	0.03	0.880
SC	MC	G*q*	107	−0.64	10	−0.72	−0.08	0.806
SC	MC	G*rw*	28	−0.73	15	−0.50	0.23	0.305
moderator: study design								
within	between	total	276	−0.63	27	−0.74	−0.11	0.510
within	between	G*kn*	123	−0.66	9	−0.79	−0.13	0.708
within	between	G*q*	109	−0.64	8	−0.69	0.05	0.868
within	between	G*rw*	37	−0.62	6	−0.83	−0.21	0.465
moderator: country of data collection								
United States	other	total*	109	−0.75	194	−0.55	0.20	0.032
United States	other	G*kn*	52	−0.81	80	−0.53	0.30	0.077
United States	other	G*q*	33	−0.76	84	−0.56	0.13	0.231
United States	other	G*rw*	17	−0.60	26	−0.71	−0.13	0.540
moderator: study scope								
small-scale	large-scale	total	107	−0.68	196	−0.61	0.07	0.623
small-scale	large-scale	G*kn*	59	−0.72	73	−0.56	0.16	0.505
small-scale	large-scale	G*q*	9	−0.49	108	−0.73	−0.24	0.364
small-scale	large-scale	G*rw*	28	−0.72	15	−0.53	0.19	0.366
moderator: year of data collection								
<2000	≥2000	total	219	−0.64	84	−0.66	−0.02	0.892
<2000	≥2000	G*kn*	86	−0.74	46	−0.62	0.12	0.600
<2000	≥2000	Gq*	107	−0.49	10	−1.02	−0.54	0.027
<2000	≥2000	G*rw*	22	−0.67	21	−0.65	0.02	0.941

*p < 0.05, **p < 0.01, ***p < 0.001.

Examining the various types of OE response formats used in the original studies, no significant differences were found in the magnitude of the gap between scores from OE and CE formats when *written short answer* items (*k* = 120; *d*_av_ = −0.66, *p* < 0.001), which are usually most similar to typical CE items, were used rather than *essay* (*k* = 32; *d*_av_ = −0.73, *p* < 0.001), *mixed OE* (*k* = 97; *d*_av_ = −0.93, *p* < 0.001), *practical task* (*k* = 11; *d*_av_ = −0.28, *p* = 0.223) and *cloze* (*k* = 42; *d*_av_ = 0.06, *p* = 0.887) item types. Further analyses with the aggregated OE format categories (*written short answer* versus *other OE types*) revealed no significant moderating effect of the type of OE format overall or for any of the individual cognitive ability categories (table 3), indicating that higher scores were obtained in CE formats as opposed to OE formats regardless of the exact OE format type that was used.

Examining the different types of CE response formats revealed that a significant amount of disparity in the difference between scores derived from OE and CE response formats depended on the extent to which the probability of guessing changed. When *true–false* items with a high guessing probability of 50% (*k* = 5; *d*_av_ = −1.51, *p* = 0.001) were used rather than *single-choice 1 out of 4 or 5 options* items with a guessing probability of 20–25% (*k* = 158; *d*_av_ = −0.60, *p* < 0.001; *d*_diff_ = 0.91, *p* = 0.032), the difference between scores from OE and CE assessments was even larger. No further disparities depending on differences in guessing probability emerged when *single-choice 1 out of 6 to 10 options* items with a guessing probability of 10–16.7% (*k* = 6; *d*_av_ = −0.54, *p* = 0.065) were applied rather than *single-choice 1 out of 4 or 5 options* items, or when *multiple-choice x out of 4 or 5 options* items with a guessing probability of 3.1–6.3% (*k* = 9; *d*_av_ = −0.29, *p* = 0.451) were used rather than *single-choice 1 out of 6 to 10 options* items. Further analyses with the aggregated CE format categories (*single-choice* versus *multiple-choice*) as the moderator revealed no significant effects of the CE format type overall or for any of the individual cognitive ability categories (table 3), demonstrating that lower scores were obtained in OE formats as compared with CE formats regardless of the exact CE format type that was applied.

The test situation's level of consequences for the test-taker (low-stakes versus high-stakes; table 3), study design (within versus between; table 3) and the scope of the original studies (small-scale versus large-scale; table 3) did not significantly moderate the difference in scores between OE and CE assessments overall or for any individual cognitive ability category, demonstrating that lower scores were reached in OE formats regardless of how important the test situation was for the test-takers, which design was used in the original studies, and regardless of the studies' scope. When the data had been collected in the United States, the difference between scores from OE and CE formats was significantly higher than for data collected in other countries overall (*d*_diff_ = 0.20, *p* = 0.032; table 3). Finally, the year of data collection (before 2000 versus in or after 2000; table 3) significantly moderated the magnitude of the difference between scores from OE and CE assessments in the cognitive ability category G*q*, indicating that significantly more CE items than OE items were correctly solved in studies examining quantitative knowledge in or after the year 2000, whereas the differences between scores from OE and CE formats in quantitative knowledge studies conducted before the year 2000 were significantly smaller (*d*_diff_ = −0.54, *p* = 0.027).

### 3.2. Results of Meta-Analyses B: correlations between scores from OE and CE formats and moderating effects of the test situation

#### 3.2.1. Descriptive statistics of Meta-Analyses B

The results of 34 primary studies with 98 effect sizes were included in the second group of meta-analyses (see electronic supplementary material, tables S2 and S5). Most of the studies had been conducted in the United States (70.6%), Canada (8.8%) and Germany (5.9%), usually with university students (61.8%) and school students (35.3%) as test-takers. Similar to Meta-Analyses A, a large majority of the authors used a within-subject design (91.2%) with non-stem-equivalent items (79.4%) in low-stakes test situations (58.8%) and small-scale assessments (82.3%). About half of the data were collected before the year 2000 (52.9%), and the number of test-takers ranged from 28 to 9314 per record. As in the first group of meta-analyses, the achievement and aptitude tests that were employed usually measured *domain-specific knowledge* (43.9%), *reading and writing* (31.7%)*, quantitative knowledge* (14.6%) and *fluid reasoning* (9.8%).

#### 3.2.2. Multi-level random effects Meta-Analyses B

The second group of multi-level random effects meta-analyses revealed a statistically significant large positive association between scores obtained in assessments with OE response formats and scores obtained in assessments with CE response formats (*r* = 0.67, *p* <0 .001; table 4; details are presented in electronic supplementary material, table S8), overall as well as separately for the cognitive ability categories G*kn* (*r* = 0.65, *p* < 0.001), G*q* (*r* = 0.85, *p* < 0.001) and G*rw* (*r* = 0.64, *p* < 0.001). This means that a large part (i.e. 41–72%) of the variance in performance on OE assessments is explained by performance on CE assessments. With respect to heterogeneity, considerable variability was revealed for all cognitive ability categories. The heterogeneity within studies was low to moderate, whereas considerable variability between studies was present for all ability categories. The Pearson's product-moment correlation between 39 effects that were available as both standardized mean differences and as correlation coefficients revealed a small non-significant relation of −0.03. This indicates that larger correlation coefficients between scores from OE and CE assessments did not coincide with smaller standardized mean differences between scores from the two formats.

**Table 4.** Results of multi-level random effects Meta-Analyses B: pooled effect sizes for the relations between scores from OE and CE response formats. *Note. k* = number of effect sizes extracted, cognitive ability: G*kn* = domain-specific knowledge, G*q* = quantitative knowledge, G*rw* = reading and writing. *r* = correlation coefficient, s.e. = standard error, 95% CI = 95% confidence interval, Cochran's *Q* and $I_{\mathrm{total}}^{2}$ = measures of heterogeneity, $I_{\mathrm{level}2}^{2}$ = heterogeneity within studies, $I_{\mathrm{level}3}^{2}$ = heterogeneity between studies. All *I*² are percentages.

cognitive ability	*k*	*r* (s.e.)	95% CI	*p*-value	Cochran's *Q*	$I_{\mathrm{total}}^{2}$	$I_{\mathrm{level}2}^{2}$	$I_{\mathrm{level}3}^{2}$
total***	98	0.67 (0.050)	[0.57, 0.76]	<0.001	*Q*(97) = 7776.83, *p* < 0.001	99.17	27.79	71.37
Gkn***	43	0.65 (0.051)	[0.55, 0.76]	<0.001	*Q*(42) = 731.07, *p* < 0.001	96.01	26.94	69.07
Gq***	24	0.85 (0.095)	[0.67, 1.00]	<0.001	*Q*(23) = 789.35, *p* < 0.001	99.16	14.77	84.39
Grw***	28	0.64 (0.104)	[0.44, 0.84]	<0.001	*Q*(27) = 2816.60, *p* < 0.001	99.60	5.24	94.36

*p < 0.05, **p < 0.01, ***p < 0.001.

#### 3.2.3. Moderation analyses B

Turning to moderating effects of the examination situation, the items’ stem-equivalency (non-stem-equivalent versus stem-equivalent; table 5) did not significantly moderate the relation between scores from OE and CE assessments, indicating a large association between scores from OE and CE response formats irrespective of stem-equivalency. The level of possible consequences of the test situation for the test-taker (low-stakes versus high-stakes; table 5) significantly moderated the relation between the scores from OE and CE tests only in the ability category G*q* (*r*_diff_ = −0.39, *p* = 0.011; table 5), indicating that the response format had a larger effect when test situations with quantitative items were personally relevant for the test-takers.

**Table 5.** Results of moderation analyses B. *Note*. *k*_1_ = number of effect sizes extracted for moderator category 1, *r*_1_ = correlation coefficient for moderator category 1, *k*_2_ = number of effect sizes extracted for moderator category 2, *r*_2_ = correlation coefficient for moderator category 2, *r*_diff_ = difference between *r*_1_ and *r*_2_, G*kn* = domain-specific knowledge, G*q* = quantitative knowledge, G*rw* = reading and writing, SA = short answer, SC = single-choice, MC = multiple-choice.

moderator category								
1	2	cognitive ability	*k*_1_	*r*_1_	*k*_2_	*r*_2_	*r*_diff_	*p*-value
moderator: stem-equivalency								
yes	no	total	86	0.62	12	0.85	0.23	0.064
yes	no	G*rw*	23	0.59	5	0.79	0.20	0.448
moderator: level of consequences of the test situation								
low-stakes	high-stakes	total	63	0.69	35	0.64	−0.04	0.587
low-stakes	high-stakes	G*kn*	19	0.70	24	0.63	−0.07	0.431
low-stakes	high-stakes	Gq*	16	0.93	8	0.53	−0.39	0.011
moderator: type of OE response format								
written SA	other OE	total***	52	0.81	46	0.51	−0.30	<0.001
written SA	other OE	G*kn*	22	0.64	21	0.67	0.03	0.771
written SA	other OE	G*rw*	9	0.83	19	0.51	−0.32	0.152
moderator: type of CE response format								
SC	MC	total	53	0.64	45	0.70	0.06	0.552
SC	MC	G*kn*	16	0.63	27	0.67	0.05	0.675
SC	MC	Gq*	14	0.73	10	1.00	0.35	0.029
SC	MC	G*rw*	21	0.65	7	0.62	−0.03	0.845
moderator: study design								
within	between	total	93	0.65	5	0.81	0.15	0.402
moderator: country of data collection								
United States	other	total	77	0.62	21	0.78	0.16	0.145
United States	other	G*kn*	35	0.64	10	0.70	0.06	0.621
United States	other	G*rw*	22	0.53	6	0.88	0.35	0.162
moderator: study scope								
small-scale	large-scale	total	71	0.62	27	0.85	0.23	0.057
small-scale	large-scale	G*kn*	35	0.65	8	0.68	0.03	0.840
small-scale	large-scale	G*q*	13	0.76	11	0.94	0.18	0.396
small-scale	large-scale	G*rw*	20	0.58	8	0.82	0.24	0.307
moderator: year of data collection								
<2000	≥2000	total	69	0.67	29	0.67	0.00	0.996
<2000	≥2000	G*kn*	23	0.63	20	0.68	0.05	0.634
<2000	≥2000	G*rw*	23	0.68	5	0.56	−0.12	0.586

*p < 0.05, **p < 0.01, ***p < 0.001.

A significantly stronger association between scores from OE and CE assessments was revealed when *written short answe*r items (*k* = 52; *r* = 0.80, *p* < 0.001), which are most similar to typical CE items, were used rather than *essay* items (*k* = 16; *r* = 0.46, *p* < 0.001; *r*_diff_ = −0.34, *p* < 0.001). Further analyses with the aggregated OE format categories (*written short answer* versus *other OE types*) yielded a significant overall moderating effect of the type of OE format, with larger correlations between scores from OE and CE assessments found when *written short answer* items were used rather than *other OE* formats (*r*_diff_ = −0.30, *p* < 0.001; table 5). For the individual cognitive ability categories, the type of OE format did not significantly moderate the association between scores obtained in CE and OE formats.

Turning to the different types of CE response formats, not enough effect sizes were reported in the original studies to examine the detailed CE formats *true–false, single-choice 1 out of 4 or 5 options, single-choice 1 out of 6 to 10 options*, and *multiple-choice x out of 4 or 5 options*. Therefore, we conducted our analyses with the aggregated CE format categories (*single-choice* versus *multiple-choice*) as the moderator. A significantly stronger correlation between scores from OE and CE response formats was revealed in studies examining quantitative knowledge with *multiple-choice* items rather than *single-choice* items (*r*_diff_ = 0.35, *p* = 0.029; table 5), whereas no significant discrepancies in the association between scores from OE with scores from CE assessments were found overall or for the cognitive ability categories G*kn* and G*rw*. Study design (within versus between; table 5), country of data collection (the United States versus other countries; table 5), study scope (small-scale versus large-scale; table 5), and the year of data collection (before 2000 versus in or after 2000; table 5) did not significantly moderate the relation between scores from OE and CE formats, indicating that the large effects were not significantly affected by design, place, scope or year of data collection.

### 3.3. Results of Meta-Analyses C: moderating effects of individual characteristics and relations between response format and school performance

#### 3.3.1. Descriptive statistics for Meta-Analyses C

The results of 91 primary studies with 337 effect sizes were included in the third group of meta-analyses (see electronic supplementary material, tables S3 and S6). The studies were conducted predominantly in the United States (52.8%), the United Kingdom (6.6%), Canada (6.6%) and Taiwan (4.4%), usually with school students (51.7%) and university students (41.8%) as test-takers. In this group of studies as well, most of the authors used a within-subject design (79.1%) with non-stem-equivalent items (64.8%) in low-stakes test situations (69.2%) and small-scale assessments (70.3%). About half of the data were collected before the year 2000 (46.2%), and the number of test-takers ranged from 15 to 191 040 per record. The achievement and aptitude tests that were employed predominantly measured *domain-specific knowledge* (45.7%), *reading and writing* (26.7%), *quantitative knowledge* (19.1%) and *fluid reasoning* (5.7%).

#### 3.3.2. Moderation analyses C

In terms of the moderating effects of individual characteristics, we hypothesized a larger difference between scores from OE and CE response formats among older groups of test-takers. Our meta-analyses indicated that age did not significantly moderate the magnitude of the gap between scores from OE and CE assessments, indicating that significantly higher scores were reached in CE response formats irrespective of the test-takers' age (table 6).

**Table 6.** Results of moderation analyses for age group (6-to-10-year-olds versus 11-to-14-year-olds versus 15-to-17-year-olds versus over 18-year-olds). *Note*. *k*_6–10_ to *k*_>18_ = numbers of effect sizes extracted for 6-to-10-year-olds, 11-to-14-year-olds, 15-to-17-year-olds, over 18-year-olds; *d*_6–10_ to *d*_>18_ = Cohen's *d* coefficients for 6-to-10-year-olds, 11-to-14-year-olds, 15-to-17-year-olds, over 18-year-olds, respectively; G*kn* = domain-specific knowledge, G*q* = quantitative knowledge, G*rw* = reading and writing.

cognitive ability	*k*_6–10_	*d*_6–10_	*k*_11–14_	*d*_11–14_	*k*_15–17_	*d*_15–17_	*k*_>18_	*d*_>18_
total	30	−0.67	127	−0.66	83	−0.67	63	−0.64
G*kn*	—	—	46	−0.70	48	−0.67	36	−0.66
G*q*	16	−0.46	72	−0.45	25	−0.70	4	−1.10
G*rw*	11	−0.63	9	−0.78	9	−0.67	14	−0.60

Difference from previous age group: *p < 0.05, **p < 0.01, ***p < 0.001.

A small pooled effect size for sex differences in favour of women was revealed in OE assessments for the ability category G*rw* (*d*_av_ = −0.14, *p* < 0.001), indicating that women tended to achieve significantly higher scores in reading and writing compared with men when OE response formats were used, whereas no significant sex differences in scores were found on OE exams overall and in the cognitive ability category G*kn.* However, in the cognitive ability category G*q* (*d*_av_ = 0.16, *p* = 0.003), men attained significantly higher scores than women when quantitative knowledge was examined in an OE format. Turning to CE assessments, overall (*d*_av_ = 0.12, *p* = 0.001) and in the cognitive ability categories G*kn* (*d*_av_ = 0.14, *p* = 0.008) and G*q* (*d*_av_ = 0.18, *p* = 0.001), small pooled effect sizes for sex differences in favour of men were obtained, indicating that men achieved significantly higher scores than women overall and in domain-specific as well as quantitative knowledge. In reading and writing, no significant sex differences were found. Contrasting CE and OE response formats, as hypothesized, significantly larger sex differences in favour of men were obtained in CE formats than in OE formats, overall (*d*_diff_ = 0.06, *p* < 0.001; table 7) and in the ability category G*kn* (*d*_diff_ = 0.12, *p* < 0.001). This means that men achieved particularly higher scores compared with women overall and when domain-specific knowledge was assessed via CE response formats, whereas smaller sex differences occurred for scores from OE response formats. For the ability category G*rw* (*d*_diff_ = 0.15, *p* < 0.001), even a significant effect favouring women revealed for scores from OE assessments.

**Table 7.** Results of analyses for sex differences in OE versus CE response formats. *Note*. *k* = number of effect sizes extracted for sex differences, *d*_OE_ = Cohen's *d* coefficient for sex differences in OE response format, *d*_CE_ = Cohen's *d* coefficient for sex differences in CE response format, *d*_diff_ = difference between *d*_OE_ and *d*_CE_, G*kn* = domain-specific knowledge, G*q* = quantitative knowledge, G*rw* = reading and writing.

cognitive ability	*k*	*d*_OE_	*d*_CE_	*d*_diff_	*p*-value
total***	183	0.06	0.12	0.06	<0.001
Gkn***	66	0.02	0.14	0.12	<0.001
G*q*	101	0.16	0.18	0.02	0.179
Grw***	12	−0.14	0.01	0.15	<0.001

*p < 0.05, **p < 0.01, ***p < 0.001.

Test anxiety had a small but significant negative effect on scores in both response formats (*r* = −0.23, *p* = 0.003; table 8), whereas risk propensity (*r* = 0.14, *p* = 0.083; table 8) slightly enhanced performance in both response formats. A significantly larger relation between achievement motivation and test scores was revealed when OE items (*r* = 0.39, *p* < 0.001) were used compared with CE test items (*r* = 0.17, *p* < 0.048; *r*_diff_ = −0.22, *p* = 0.031; table 8). In an exploratory moderator analysis, we found that response format significantly moderated the relation between test scores and school performance. Overall, significantly larger correlation coefficients were revealed when CE items (*r* = 0.38, *p* < 0.001) were used compared with OE items (*r* = 0.27, *p* < 0.001; *r*_diff_ = 0.11, *p* = 0.002; table 8). In the cognitive ability field G*kn*, too, larger relations between test scores and school performance emerged when CE items (*r* = 0.42, *p* < 0.001) were used rather than OE items (*r* = 0.28, *p* < 0.001; *r*_diff_ = 0.14, *p* = 0.001; table 8).

**Table 8.** Results of moderation analyses for test anxiety, risk propensity, motivation and school performance in OE versus CE response formats. *Note. k* = number of effect sizes extracted, *r*_OE_ = correlation coefficient in OE response format, *r*_CE_ = correlation coefficient in CE response format, *r*_diff_ = difference between *r*_OE_ and *r*_CE_, G*kn* = domain-specific knowledge.

cognitive ability	*k*	*r*_OE_	*r*_CE_	*r*_diff_	*p*-value
moderator: test anxiety					
total	9	−0.23	−0.23	0.00	0.962
G*kn*	4	−0.19	−0.19	0.00	0.949
moderator: risk propensity					
total	8	0.14	0.14	0.00	0.903
moderator: motivation					
total*	5	0.39	0.17	−0.22	0.031
moderator: school performance					
total**	17	0.27	0.38	0.11	0.002
Gkn**	12	0.28	0.42	0.14	0.001

*p < 0.05, **p < 0.01, ***p < 0.001.

## 4. Discussion

We conducted three groups of multi-level random effects meta-analyses to estimate mean effect sizes for the differences and relations between scores from achievement and aptitude measures in OE and CE response formats. Furthermore, we aimed to examine moderating effects of the examination situation and of individual characteristics of the test-takers. Considering all available data, the results of 102 primary studies with 392 effect sizes revealed large positive correlations between scores from CE and OE assessments with medium to large negative pooled effect sizes for the difference between scores from the two response formats, indicating that the same concepts have usually been measured by the two formats, but, in general, significantly higher scores have been obtained on CE exams, as hypothesized, and supporting previous findings (e.g. [25,33]). The uncorrected correlation coefficient of 0.67 revealed in our meta-analyses is as high as the relation reported by Rodriguez [25] in his previous meta-analysis. Interestingly, stronger relations between scores from OE and CE assessments did not coincide with smaller standardized mean differences between scores from the two formats. As the first indicator may be interpreted as capturing the items’ reliability to assess individuals’ high as well as low performance across item formats, the second addresses the absolute differences that could also be a result of, for example, item difficulty differences in the different versions.

Regarding potential moderating effects originating in the examination situation, the stem-equivalency of the items significantly influenced the difference between scores from OE and CE assessments. When the items in the two response formats had the same item stem, the results were much more similar than when tasks were presented with different stems, as hypothesized and reported previously by, for example, Rodriguez [25] and In'nami & Koizumi [33]. Overall and especially for domain-specific knowledge, when the only difference between the OE and CE versions of an item was that response options were provided for the latter, construct equivalence was revealed to be larger between the two response formats. The severity of the possible consequences of the test situation for the test-taker was associated with the size of the relation between scores from OE and CE assessments when quantitative knowledge was measured. We hypothesized that impairing factors, such as test anxiety, would have stronger effects on performance in high-stakes test situations that could have severe consequences for the test-taker, as has been reported in the literature (e.g. [34,35]). Our data for this moderator indeed yielded significantly stronger relations between scores from OE and CE assessments in low-stakes test situations assessing quantitative knowledge, indicating that impairing and enhancing factors exhibited a larger effect on performance when test applications had potential consequences for the test-takers.

The most frequently used type of OE response format in the original studies was *written short answer*. When *essay* tasks were presented instead of *written short answer* items, significantly smaller associations between scores from OE and CE assessments were found. Analyses with the aggregated OE format categories (*written short answer* versus *other OE types*) revealed that, in line with our hypothesis, scores from different response formats were related the most when *written short answer* items, which are usually most similar to typical CE items, were used (e.g. [25,38]) rather than other OE item types. Taking a closer look at the different types of CE response formats that were presented in the original studies revealed that the magnitude of the guessing probability moderated the differences and relations between scores from OE and CE items. Response format had a larger impact on *true–false* items compared with CE items with lower guessing chances. Turning to analyses with aggregated CE format categories (*single-choice* versus *multiple-choice*), our assumption that *single-choice* items with higher guessing odds than *multiple-choice* tasks would exhibit smaller relations between scores from OE and CE assessments, also proposed by Kubinger & Gottschall [39], was revealed to be applicable only to quantitative knowledge in our meta-analyses. This indicates that higher odds of guessing in the CE format might be especially helpful in ability fields covering maths or statistics skills, possibly resulting in stronger relations between scores from different response formats when guessing can be prevented to a greater degree.

Study scope and design were not significant moderators of the differences and the relations between scores retrieved from CE and OE assessments, showing that higher scores were reached in the CE format, regardless of whether the items were presented in small-scale or large-scale studies and in within- or between-designs. As almost half of the integrated data were collected in the United States, we aimed to examine potential moderating effects of the originating country. The results indeed showed that scores from OE and CE measures differed to a larger extent when the data were collected in the United States, perhaps indicating that the tradition of how to create items in OE and CE response formats in the United States is somewhat different from in other countries. When quantitative knowledge was measured, even the year of data collection had a significant moderating effect on the magnitude of differences between scores from OE and CE exams. In or after the year 2000, significantly more CE items than OE items were solved in maths or statistics assessments, perhaps indicating that the way CE quantitative knowledge items are created has changed over the years.

Further, we examined possible moderating effects of individual characteristics in our analyses of the results of 91 primary studies with 337 effect sizes. Concerning the hypothesized influence of age on the difference between OE and CE scores, the results revealed no moderating effect of increasing age. Although some aspects of personality that have previously been addressed as relevant with reference to response format (e.g. test anxiety, risk propensity, achievement motivation) are known to generally develop and differentiate further during puberty (e.g. [41,57,62,63]), the results of older individuals did not exhibit larger differences between scores from varying response formats than the results of younger test-takers. With respect to sex differences in OE and CE response formats, our hypotheses were supported by the findings: significantly larger sex differences in favour of men were found in CE assessments overall and when domain-specific knowledge was assessed, supporting the assumption that, overall, men are more prone to guessing and successfully using test-taking strategies in examination situations (e.g. [32,58,59]). By contrast, the results of OE exams revealed smaller sex differences, and women even outperformed men on reading and writing assessments when OE response formats were used. In quantitative knowledge, however, men not only significantly outperformed women when CE items were used, but also when OE items were presented.

Even though we expected test anxiety to be more strongly related to performance on OE items and risk propensity to performance on CE items as proposed in the literature (e.g. [26,42,45,46]), test anxiety had a small significant debilitating effect, and risk propensity a small enhancing influence on scores in both response formats. As achievement motivation has been linked to more efficient use of test-taking strategies (e.g. [48]), and these have been revealed to be especially performance-enhancing in CE assessments (e.g. [13,49]), we hypothesized motivation to be more strongly related to performance on CE items compared with OE items. By contrast, the analyses revealed that achievement motivation had a significantly larger positive impact on scores when OE items were used rather than CE assessments. This finding may indicate that giving one's best effort may be particularly performance-enhancing when responses have to be constructed. This could be possibly explained by the higher demands on motivation involved in providing longer, more elaborate, more detailed or creative answers to which raters may assign better scores. In summary, test-takers possessing lower levels of anxiety and higher levels of risk propensity obtained higher scores in both response formats, whereas test-takers with higher levels of achievement motivation scored particularly high in OE formats. Finally, analyses revealed significantly higher criterion validity scores for academic success for achievement and aptitude tests with CE response formats compared with OE formats.

## 5. Implications and limitations

In the presented meta-analyses, we comprehensively and systematically investigated the effects of various open and closed response formats on the results of achievement and aptitude tests aimed at assessing a large number of cognitive abilities, skills and knowledge, including varying characteristics of examination situations and test-takers as moderators. With the largest aggregation of studies on this topic so far, this paper extends the previous literature and offers more precise information and a better understanding of the sizes and directions of format effects. As standardized achievement and aptitude assessments are basic elements of individuals' everyday lives and may have tremendous consequences for educational, academic and professional success, the results of our meta-analyses have obvious methodological implications for a large number of people who are developing and implementing tests and for the people who are responsible for evaluating individuals and organizations, for example, students, instructors and curriculum leaders from primary to post-secondary education; trainees, job applicants and human resource managers in all professional fields; educational and psychological researchers; and policymakers—in short, all those involved in assessment. The results confirm that, when planning an assessment, it is crucial to consider not only *what* will be tested but also *how* it will be tested. If individuals of different sexes, social groups, or ethnicities are tested, person-related bias should be avoided. Furthermore, if more than one method of item presentation is possible, the institutions that base decisions on test scores have the responsibility to consider the results of the current study when deciding which methodological approach to employ.

In general, the results of our meta-analyses confirmed several earlier individual research results claiming that both CE and OE response formats have strengths and weaknesses that either support or oppose their use with reference to certain aims (e.g. [13–18]). The benefits of CE items (i.e. their objectivity, economy and efficiency of scoring) make them a very important and convenient assessment tool. The fact that a larger number of items can be presented in a shorter amount of time increases their reliability. On the other hand, the greater opportunity to use potentially biasing, construct-irrelevant test-taking strategies (e.g. guessing) in CE formats may systematically benefit those who are willing to use such strategies at all, hence threatening test fairness. CE items may be answered using basic recognition without necessarily requiring individuals to understand the nature of a posed problem. Some researchers argue that high scores on CE items do not necessarily indicate that test-takers have mastered appropriate strategies to solve problems in real life. Hence, the need for productive retrieval processes and therefore the possibility of assessing higher-level cognitive processes while covering a broader range of skills and abilities, in turn increasing reliability, might represent an advantage of OE formats over CE formats (e.g. [94,95]). Other researchers postulate that the main additional value of OE items over CE formats is that they can better assess value judgements and the combination or generation of ideas (e.g. [17,96,97]). The disadvantages of OE formats obviously involve the often greater effort and subjectivity involved in the scoring process. Furthermore, responding to some OE items might require a higher level of linguistic skills among both test-takers and instructors (e.g. [32,98]). Given that linguistic and verbal skills might affect scores in, for example, mathematics or content-specific knowledge raises concerns about not only validity but also test fairness, as non-native speakers or people with dyslexia might be systematically disadvantaged by deficits in construct-irrelevant abilities (e.g. [99]).

On the basis of these results, we recommend that test administrators carefully and individually choose the most suitable response format depending on the specified requirements and the population targeted by a test application. If there is no clear evidence that a specific response format is better for the given population, a multi-method approach combining the two response formats might be advisable. Preparing test-takers for the possibility that assessments may include not only CE but also OE items might lead them to study for exams by developing a conceptual understanding rather than by memorizing facts by rote (e.g. [100]). Encoding information conceptually will enable test-takers to handle both response formats, whereas memorizing by rote will only prepare them for CE assessments. To prepare test-takers for the two kinds of assessments equally, however, requires instructors who are able to ensure that the nature of a problem is actually understood and who are able to teach strategic learning as well as problem solving, for example, through regular discourse and training in OE performance tasks (e.g. [65,100,101]). Curriculum leaders and policymakers may support this process by setting up and coordinating educational programmes with a focus on teaching problem solving and critical thinking skills.

Another important implication for those who design and implement assessments is given by our finding that performance test scores were most similar when stem-equivalent items were used throughout the different formats. This indicates that differences in scores may arise primarily from different content being assessed with different formats. Therefore, it is crucial to consider exactly what knowledge or ability should be assessed, rather than what can be assessed conveniently. As a second issue, more work should be invested in designing objective scoring guidelines when developing OE items in order to reduce the effort involved in grading. Items should be independent of each other to avoid consequential errors that decrease measurement quality [98]. Due to ongoing technical advances (e.g. automated text analysis), the scoring of OE tests may be conducted more economically even in large-scale assessments.

As test fairness currently represents one of the most central concerns and critical issues in psychological and educational assessment (e.g. [11,102]), our findings concerning the effects of test-taker characteristics may contribute to the question of the emergence of test bias. For example, our finding that the scores obtained from OE exams revealed smaller advantages for men than found with CE exams might not be very large but may indicate that the response format can lead to significant changes in the proportions of women and men earning certain grades, passing tests, getting admitted to programmes or being selected for employment. However, as our results concerning individual characteristics (e.g. test anxiety, risk propensity and achievement motivation) were based on a small sample of studies that included fewer than 10 effect sizes each, further research is required here. Future research should address the effects of response format on psychometric properties and the fairness of achievement and aptitude tests by including not only the potential effects of the aforementioned individual characteristics in more detail. Future studies should include further characteristics that have the potential to impact test performance, such as conscientiousness, self-confidence, self-efficacy, neuroticism, agreeableness or possible differential effects of extrinsic and intrinsic motivation.

Methodological analyses of the relations between effect sizes and their variance partially indicated that funnel asymmetry was at play. Besides the high level of heterogeneity in published studies, publication bias may also serve as an explanation for such a result. However, in contrast to the expected effect that smaller studies would show larger effects if publication bias existed, the opposite tendency was revealed: studies based on higher precision tended to indicate larger effects than smaller studies. Effects were especially heterogeneous in studies with high precision. This result is also contrary to a scenario in which publication bias is the driving force behind funnel asymmetry. Taking these results into consideration, we suggest that the high level of heterogeneity in the included studies may have led to the given funnel asymmetry (see electronic supplementary material, figure S1). Significant statistical results were facilitated through large sample sizes and thus high power in most of the included studies. However, because a substantial amount of the data was extracted from studies investigating effects other than response formats as a main research question, publication bias seems less likely. Nevertheless, risks of bias may stem from other conditions: most primary studies did not include random sampling, random allocation of participants to the formats, randomization of the items, blinding or attrition management with regard to the research question about response formats (see electronic supplementary material, figures S2 and S3 for our risk-of-bias assessment according to the *Cochrane handbook for systematic reviews of interventions*, v. 5.1.0; [103]). Furthermore, although corrections for correlation coefficients exist to control for some types of methodological artefacts, most of the original studies included here did not report corrected estimates or sufficient information to correct for potentially biased effects. Nevertheless, due to study artefacts, the reported effect sizes within this study are probably underestimations and are likely to represent the lower bounds of validity. Future research should, therefore, include a large number of high-powered and preregistered studies focusing on the specific research question of response format effects. We also encourage future researchers in the field to publish all relevant data that allow for more detailed analyses to avoid potential biases.

## 6. Conclusion

With these meta-analyses, we aimed to uncover the effects of response format on test results and shed light on the question of the construct equivalence of examinations with different formats—for test-takers possessing different characteristics. Even though strong relations were revealed between the results of the two most popular response formats used in achievement and aptitude testing (i.e. OE and CE), the medium-sized to strong negative pooled effect sizes identified for the differences between scores from these two response formats strengthen the doubts about their construct equivalence postulated earlier (e.g. [26–28]). In our meta-analyses, we identified the stem-equivalency of the items, the types of OE and CE response formats, the severity of possible consequences, as well as the year and the place of data collection as test features originating in the test situation that may contribute to the emergence of bias. Furthermore, we uncovered diminishing effects of some of the test-takers’ construct-irrelevant individual characteristics (e.g. sex) on the fairness of achievement and aptitude tests in varying response formats. Being aware of the item features and psychological characteristics that are irrelevant to the measured construct but may be causing systematic variance in test performance between individuals and groups may help practitioners and researchers to improve not only test fairness but also validity and measurement precision in future achievement and aptitude assessments.

## Data accessibility

Datafiles, R codes and codebook for this meta-analysis can be found in the Open Science Framework (https://osf.io/vry9f/?view_only=fca6caab4b3341cb9b5b6dccfa576859) and additional tables and figures are provided in electronic supplementary material [104].

## Footnotes

1 We divided by ‘SD_pooled_’ instead of ‘2’ because this also works with unequal sample sizes per group.
